# Inhibin β-A (INHBA) induces epithelial–mesenchymal transition and accelerates the motility of breast cancer cells by activating the TGF-β signaling pathway

**DOI:** 10.1080/21655979.2021.1957754

**Published:** 2021-08-04

**Authors:** Yingying Yu, Weiwei Wang, Wenying Lu, Wei Chen, Anquan Shang

**Affiliations:** a Department of Obstetrics and Gynecology, the International Peace Maternity & Child Health Hospital, Shanghai Jiao Tong University School of Medicine, Shanghai, P. R. China; b Shanghai Key Laboratory of Embryo Original Diseases, Shanghai, China; c Shanghai Municipal Key Clinical Specialty, Shanghai, China; d Department of Pathology, Tinghu District People’s Hospital of Yancheng City, Yancheng, Jiangsu, China; e Department of Emergency, Tongji Hospital, Tongji University, School of Medicine, Shanghai, China; f Department of Laboratory Medicine, Shanghai Tongji Hospital, Shanghai, China

**Keywords:** Breast cancer, INHBA, TGF-β, EMT, cancer cell invasion

## Abstract

Accumulating evidence indicates that INHBA (Inhibin β-A, a member of the TGF-β superfamily) functions as an oncogene in cancer progression. However, little is known as to how INHBA regulates the progression and aggressiveness of breast cancer (BC). This study explored the function and underlying mechanism of INHBA in epithelial–mesenchymal transition (EMT) of BC cells. INHBA expression in BC cell lines was measured using RT-qPCR and Western blot. The would-healing and transwell migration assays were used to investigate the effect of INHBA overexpression or silencing on BC cell motility. Moreover, the expression levels of EMT-related genes were quantified after overexpressing or silencing of INHBA. Based on published dataset, INHBA was significantly upregulated in BC tissues compared to the adjacent normal tissues. A higher level of INHBA expression was also correlated with a poor survival in BC patients. In addition, *in vitro* study showed that INHBA played an indispensable role in promoting BC cell proliferation and invasion. Mechanistically, INHBA induced epithelial–mesenchymal transition (EMT) and accelerated the motility of BC cells by activating TGF-β-regulated genes. In conclusion, INHBA plays a functional role in supporting EMT phenotype of BC cells, and it may serve as a diagnostic biomarker and a potential therapeutic target for BC treatment.

## 1. Introduction

Breast cancer (BC) is the most commonly diagnosed cancer in female [1]. Due to recent advances in early screening and therapy, the survival rates of breast cancer have been increasing [2]. The decline of overall mortality is attributed to early screening program and a better understanding of pathogenesis of the disease [3,4]. The efforts in delineating molecular signatures of BC also provided useful guidance into the optimization of treatment plan. Based on the genomic signatures, breast cancer is categorized into estrogen receptor (ER) positive, HER2 amplified, and triple negative subtype [5]. Therefore, exploring the molecular fingerprints of BC can be potentially used for patient stratification and personalized medicine.

Inhibin βA (INHBA) is a member of TGF-β superfamily, which forms a disulfide-linked homodimer called activin A [6,7]. It has been demonstrated that BC expresses activin A mRNA and proteins, and activin A promotes the EMT and metastatic growth of BC cells through SMAD signaling pathway [8,9]. A recent study showed that activin A overexpression promotes BC metastasis by activating Smad2 and inducing IL13Ra2 expression [10]. INHBA overexpression has been correlated with aggressive phenotype in different cancers, including colorectal cancer, lung adenocarcinoma, esophageal adenocarcinoma, ovarian cancer, and gastric cancer [11–15]. INHBA overexpression is also associated with a poor prognosis in gastric and lung cancer [14,16]. Seder et al. demonstrated that INHBA overexpression enhances cell proliferation in lung and esophageal cancer [11,14], and Li et al. showed that INHBA is upregulated in advanced and high-grade serous ovarian cancer and induces stromal fibroblast activation to support tumorigenesis [15]. They further revealed that INHBA knockdown hampers the tumorigenesis in an ovarian cancer xenograft model by suppressing the activation of stromal fibroblast. However, the role of INHBA in BC tumorigenesis and metastasis remains to be elucidated.

Currently, a growing body of studies focus on unveiling the molecular and cellular mechanisms underlying the invasion and motility of cancer cells [17]. Epithelial–mesenchymal transition (EMT) has been recognized as a critical process in embryonic development, wound healing, cancer cell migration and the metastatic progression of carcinomas [18–20]. During EMT, epithelial cells which originally adhere on a basement membrane gradually lose its epithelial polarity and gain mesenchymal phenotype of enhanced migratory capability [20]. The key events involved in EMT include loss of apical-basal polarity, cytoskeleton reorganization, and the downregulation of epithelial gene and protein [19]. Several biomarkers have been identified as indicators for EMT, such as E-cadherin, ZO-1, Fibronectin, Snail, Slug, Twist and DDR-2 [17]. A study by Bashir et al. demonstrated that activin A promotes EMT in BC, which contributes to the invasion and metastasis of BC cells [21]. Meanwhile, other studies also highlighted the role of TGF-β in mediating cancer progression through the induction of EMT [22,23]. However, the direct involvement of INHBA in EMT event has not been reported in BC.

In this study, we aim to explore the function and underlying mechanism of INHBA in epithelial–mesenchymal transition (EMT) of BC cells. Our results showed that INHBA played an indispensable role in promoting BC cell proliferation and invasion. Mechanistically, INHBA induced epithelial–mesenchymal transition (EMT) and accelerated the motility of BC cells by activating TGF-β-regulated genes. Our study provides in-depth understanding of how INHBA regulates the aggressiveness of BC cells. These findings indicate that INHBA may serve as a diagnostic biomarker and a potential therapeutic target for BC treatment.

## 2. Materials and methods

### 2.1. BC cell culture

Four BC cell lines, including MCF‐7, MDA‐MB‐436, MDA‐MB‐231 and BT549, and a normal epithelial cell, MCF-10A, were purchased from ATCC. Royal Park Memorial Institute (RPMI)-1640 medium (30–2001, ATCC, Manassas, VA) was used as the base medium. Ten percent fetal bovine serum (FBS), streptomycin (100 mg/ml) and penicillin (100 U/ml) were used as supplements in base medium. The cell culture medium was changed every 2 days. A humidified incubator was used for cell culture at 37°C with 5% CO_2_.

### 2.2. Cell transfection

Cells were seeded in 6-well plates at a density of 5 × 10^5^ cells/well. When cells reached 80% confluence, plasmids were transfected to BT549 and MCF-7 cells using Lipofectamine 2000 (11,668,019, Invitrogen) according to the manufacturer’s instructions. The plasmid containing human INHBA cDNA (OriGene, Beijing, China) or an empty vector as negative control (OriGene, Beijing, China) was transfected to MCF-7 cells. The plasmid containing sh-INHBA (OriGene, Beijing, China) or negative control sh-NC (OriGene, Beijing, China) was transfected to BT549 cells. shRNA sequence targeting human INHBA were shown as below: INHBA#1: 5ʹ-CCATGTCCATGTTGTACTA-3ʹ, INHBA#2: 5ʹ-GCAACAAATTGATGAGCAA-3ʹ, and control sequence: 5ʹ-CAACAAGATGAAGAGCACCAA-3ʹ. qRT-PCR was performed to confirm the overexpression or knockdown efficiency 48 hours after transfection.

### 2.3. Isolation of total RNA and qRT-PCR

Total RNA was extracted from cells using TRIzol RNA Isolation Reagents (15,596,026, ThermoFisher Scientific, Rockford, IL). The extracted total RNA was dissolved in DEPC water and its concentration was measured with NanoDorp. Five micrograms of total RNA was used for reverse-transcription into cDNA using LunaScript® RT SuperMix Kit (E3010, BioLabs, MA,USA). The resulted cDNA was analyzed on a 7500 Real-Time PCR System (Applied Biosystems/Life Technologies, Carlsbad, CA, USA) using SYBR premix EX TAQ II kit (RR820B, Takara, Dalian, China). Finally, the 2–∆∆Ct method was used to analyze the relative expression level and GAPDH was used as the internal reference gene. All primer sequences were synthesized and purchased from Shanghai Sangon Biotechnology Co., Ltd. (Shanghai, China): The primer sequences are listed in Table 1.

**Table 1.** Primer sequences used for qRT-PCR in our study

Genes	Sequences
INHBA	F: 5ʹ-CCTCCCAAAGGATGTACCCAA-3ʹ
	R: 5ʹ -CTCTATCTCCACATACCCGTTCT-3ʹ
E-cadherin	F: 5′-CGAGAGCTACACGTTCACGG-3′
	R: 5′-GGGTGTCGAGGGAAAAATAGG-3′
ZO-1	F: 5′-CAACATACAGTGACGCTTCACA-3′
	R: 5′-CACTATTGACGTTTCCCCACTC-3′
CK-8	F: 5′-CAGAAGTCCTACAAGGTGTCCA-3′
	R: 5′-CTCTGGTTGACCGTAACTGCG-3′
CK-18	F: 5′-GGCATCCAGAACGAGAAGGAG-3′
	R: 5′-ATTGTCCACAGTATTTGCGAAGA-3′
CK-19	F: 5′-AACGGCGAGCTAGAGGTGA-3′
	R: 5′-GGATGGTCGTGTAGTAGTGGC-3′
ZEB1	F: 5′-GATGATGAATGCGAGTCAGATGC-3′
	R: 5′-ACAGCAGTGTCTTGTTGTTGT-3′
Snail	F: 5′-TCGGAAGCCTAACTACAGCGA-3′
	R: 5′-AGATGAGCATTGGCAGCGAG-3′
Slug	F: 5′-CGAACTGGACACACATACAGTG-3′
	R: 5′-CTGAGGATCTCTGGTTGTGGT-3′
N-cadherin	F: 5′-TCAGGCGTCTGTAGAGGCTT-3′
	R: 5′-ATGCACATCCTTCGATAAGACTG-3′
Vimentin	F: 5′-GACGCCATCAACACCGAGTT-3′
	R: 5′-CTTTGTCGTTGGTTAGCTGGT-3′
Fibronectin	F: 5′-CGGTGGCTGTCAGTCAAAG-3′
	R: 5′-AAACCTCGGCTTCCTCCATAA-3′

### 2.4. CCK-8

Cells were cultured in 96-well plates at a density of 3500 cells/well. Then, cells were transfected with indicated plasmids and incubated for 1, 2, 3 and 4 days. Cell viability was quantified using Cell Counting Kit-8 (CCK-8) assay (CK04, Dodinjo, Japan). A quantity of 10 μL CCK8 reaction solution was added to the cell culture at indicated time point and the cells were incubated for 1 hour in a humidified cell culture incubator. The light absorption value (OD value) in each condition was captured at 450 nm wavelength on Synergy H1 microplate reader (Winooski, Vermont, USA).

### 2.5. Cell invasion and migration analysis

Cell migration ability was examined using wound healing assay 48 hours after transfection. The cells were cultured on 12 well plates till 80% confluence. A straight scratch was created using a sterile tip and detached cells in the medium were removed by gentle wash. After 48 hours, the cell migration was imaged under Leica AM6000 microscope.

Cell invasion ability was evaluated by transwell assay using QCM Endothelial Cell Invasion Assay Kit (ECM210, Sigma). Transwell upper chamber was coated with Matrigel for invasion assay. Cells were inoculated into the transwell upper chamber in serum-free medium and 500 μL of medium containing 10% FBS was added to the lower chamber. After 10 hours, culture medium was discarded and the cells were fixed with 4% paraformaldehyde at room temperature for 10 mins and stained with 0.5% crystal violet (115,940, Sigma, Germany) for 20 mins. Cells were photographed under Leica AM6000 microscope and the number of invading cells was counted.

### 2.6. Western blot

RIPA buffer (R0278, Sigma) was used for cells lysis and the cell lysate was centrifuged for 15 min at 13,000 r/min, the supernatant was collected for protein measurements. The concentration of total protein was measured by Bicinchoninic Acid (BCA) Kit (71,285 M, Sigma), and 50 µg of protein was separated on 10% SDS-PAGE gel for electrophoresis and then transferred to PVDF membranes. The membranes were blocked using nonfat milk at room temperature (RT) for 1 hours, followed by incubation with primary antibodies at 4°C overnight. After three washes with TBST buffer, the membrane was further incubated with the HRP-conjugated secondary antibody at RT for 2 hours. All antibodies (INHBA, E-cadherin, ZO-1, CK-8, CK-18, CK-19, ZEB1, Snail, Slug, N-cadherin, Vimentin and Fibronectin, and HDRP-conjugated secondary antibodies) were purchased from Abcam (Cambridge, MA, USA).

### 2.7. In vivo experiments

Six-week-old RAG1-deficient mice (n = 24) were equally separated into four groups (n = 6 in each group) randomly. Each mouse in four groups was inoculated subcutaneously with 1 × 10^5^ cells (MCF-7) transfected with empty vector, INHBA cDNA, sh-INHBA, or sh-NC in the left flank. The tumor volume was measured every 5 days after the inoculation. The tumor volume was calculated as volume (mm^3^) = largest diameter × (smallest diameters)^2^ × 0.52. After 35 days, the mice were euthanized, and the tumors were removed and weighted. The animal study was approved by the Ethics Committee of Shanghai Jiao Tong University School of Medicine (TJ-2015/H2U).

### 2.8. Immunohistochemistry (IHC)

Immunohistostaining was performed on 4-mm sections of formalin-fixed paraffin-embedded (FFPE) tumor tissue using VENTANA BenchMark Special Stain platform (Roche, Indianapolis, IN, USA). Sections were deparaffinized and hydrated in three washes of xylene for 5 min each, in two washes of 100% ethanol for 10 min each, in two washes of 95% ethanol for 10 min each, and in two washes in dH2O for 5 min each. Antigen unmasking was performed by heating the section in a microwave submersed in 1X citrate unmasking solution (SignalStain® Citrate Unmasking Solution (10X) (#14,746), Cell Signaling Technologies) until the boiling is initiated, which was followed by 10 min at a sub-boiling temperature (95°–98°C). Sections were cooled on bench top for 30 min. After three times washes in TBST buffer, the section was blocked for 1 hour at room temperature in TBST with 5% Normal Goat Serum, and then incubated with primary antibody Ki-67 (Cell Signaling Technologies #12202S) and INHBA (ab56057) diluted in SignalStain® Antibody Diluent (#8112) in 1:500 overnight at 4°C. Then, antibody solution was removed and the section was washed three times using TBST buffer. After incubated with HRP-conjugated secondary antibody, the section was soaked with 1–3 drops SignalStain® Boost Detection Reagent (HRP, Rabbit #8114, Cell Signaling Technologies) and incubated in a humidified chamber for 30 min at room temperature. A quantity of 100–400 µl SignalStain® substrate (#8059, Cell Signaling Technologies) was added to each section for 5 minutes. The section was washed in dH2O two times for 5 min each and then dehydrated. Section was mounted with coverslips using the mounting medium (#14,177, Cell Signaling Technologies) before imaging.

### 2.9. Statistical analysis

Each experiment was repeated at least 3 times. All statistical analyses were performed using SPSS 20.0 software and GraphPad Prism (GraphPad, San Diego, CA). Two tails student's t-test and one-way ANOVA plus Tukey’s post hoc test were used to analyze the statistical differences. Pearson Correlation analysis was used to determine the correlation between INHBA expression and other EMT-related genes (TGF-β1, Smad2, Smad7, Snail and Slug, and CK-19). *P* < 0.05 was considered as statistically significant.

## 3. Results

In this study, we explored the role of INHBA in EMT and the tumorigenesis of BC cells. We showed that INHBA was upregulated in BC patients and BC cells, and a high level of INHBA expression was associated with a poor prognosis. Knocking down INHBA inhibited BC cell growth and invasion, and INHBA overexpression enhanced the proliferation of BC cells. Furthermore, INHBA silencing enhanced the expression of epithelial markers (E-cadherin, ZO-1, CK-8, CK-18 and CK-19) and downregulated the expression of mesenchymal markers (ZEB1, Snail, Slug, N-cadherin, Vimentin and Fibronectin). We further demonstrated that INHBA knockdown negatively regulated TGF-β signaling pathway by downregulating the expression of TGF-β1, p-Smad2, p-Smad3. Finally, the role of INHBA in BC cell tumorigenesis was validated in a xenograft mouse model.

### 3.1. Overexpression of INHBA in BC patients

**Figure 1.**
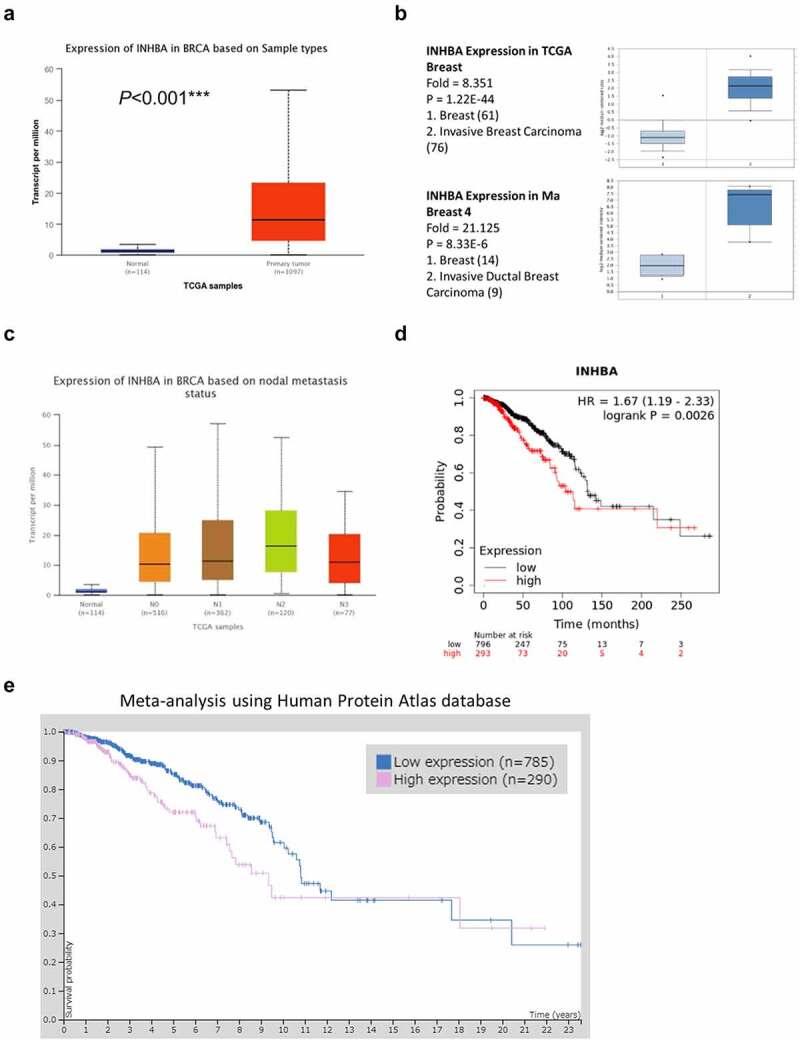
INHBA overexpression is associated with a poor survival rate in BC. (a) Upregulation of INHBA in BC tissue from TCGA database. (b) Upregulation of INHBA in two sub-types of BC as compared to normal tissues, using Oncomine database. (c) INHBA overexpression in BC patients with lymph node metastasis (N stage indicates the level of lymph node metastasis; the higher the number, the more metastasis in lymph node). Data were retrieved from TCGA database. (d) INHBA overexpression is correlated with a worse prognosis in BC patients (TCGA database). (e) BC data from Human Protein Atlas database was used analyze the correlation between the protein level of INHBA and the prognosis of BC patients (*P* = 0.0041)

The genomic and molecular profile of INHBA related to BC was retrieved from TCGA (https://www.cancer.gov/about-nci/organization/ccg/research/structural-genomics/tcga). To perform an in-depth analysis of the TCGA data, an online tool UALCAN (http://ualcan.path.uab.edu) was used to compare the relative expression of INHBA across tumor and normal tissues [24]. The result revealed that INHBA expression was significantly upregulated in primary BC patients (Figure 1a). Next, we further confirmed the INHBA overexpression in two sub-types of BC, invasive breast carcinoma and invasive ductal breast carcinoma, using Oncomine database (Figure 1b). Furthermore, our analysis also demonstrated that INHBA was upregulated in patients with lymph node metastasis at N1, N2, and N3 stage (Figure 1c). The expression level of INHBA was significantly higher in samples with the lymph node metastasis when compared to the samples showing no lymph node metastasis (N0 vs N2: *P* = 2.440900E-03) (Figure 1c). In addition, Kaplan–Meier analysis (https://kmplot.com/analysis/) was used to compare the survival rate of BC patients with low and high INHBA expression. Patients with high INHBA expression were associated with a poorer prognosis (*P* < 0.001, Figure 1d). Furthermore, the Human Protein Atlas database (https://www.proteinatlas.org) was also used to analyze the correlation between protein level of INHBA and the survival of BC patients. Similarly, INHBA protein level seemed to be an unfavorable prognostic factor due to a poorer overall survival in patient with high INHBA expression (*P* = 0.0041, Figure 1e). Together, these analyses indicate that INHBA might act as a tumor promoter in BC cells.

### 3.2. INHBA silencing inhibits BC cell growth and invasion

**Figure 2.**
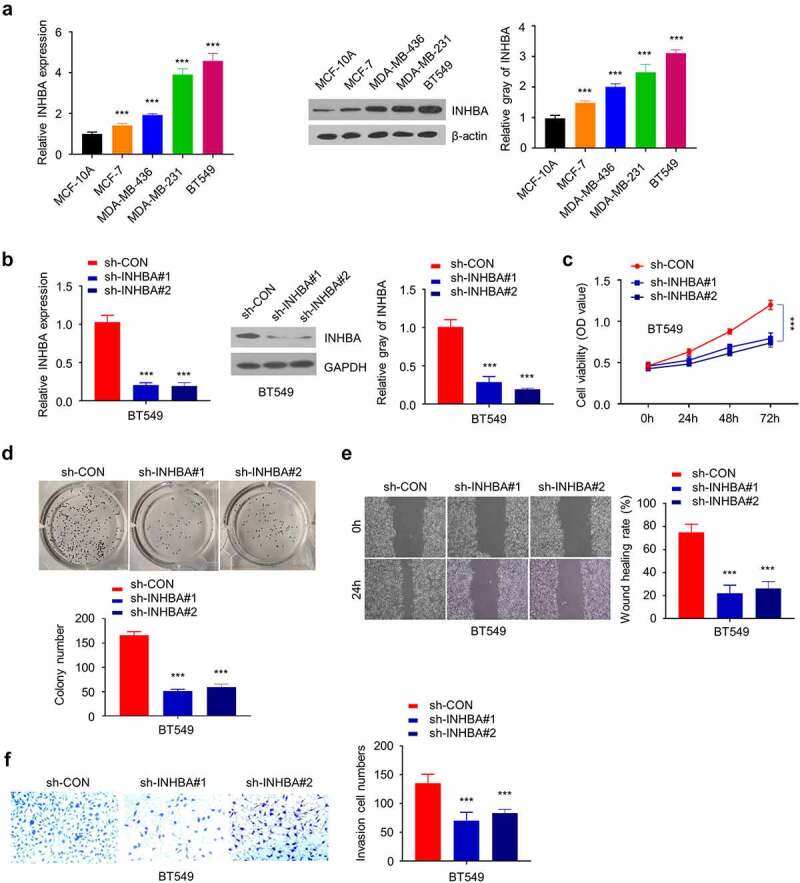
The INHBA is upregulated in BC cells and facilitates cell growth. (a) The INHBA overexpression in four BC cell lines was measured by RT-qPCR and Western blot. β-actin was used as the loading control. (b) The efficiency of INHBA knockdown in BT549 cells by sh-INHBA#1/2 or sh-NC vectors was assessed by RT-qPCR and Western blot. GAPDH was used as a loading control in Western blot. (c) CCK-8 assay and (d) colony formation assay were performed to examine the cell proliferation of BT549 cells after the transfection of shRNA. (e) The migration of transfected cells was examined by wound healing assay. (f) The invasion ability of transfected cells was determined by transwell assays (×200 magnification). Data are summary of 3 independent experiments (n = 3). The error bars are defined as s.d. *, *P* < 0.05, **, *P* < 0.01, and ***, *P* < 0.001

To study the function of INHBA in BC cells, the mRNA and protein level of INHBA in four BC cell lines (MCF-7, MDA-MB-436, MDA-MB-231 and BT549) was compared to that in a non-tumorigenic epithelial cell-line MCF-10A by RT-qPCR and western blot. The results showed that the gene and protein levels of INHBA were significantly upregulated in four BC cell lines (*P* < 0.001, Figure 2a). MDA-MB-231 and BT549 cell lines showed a higher INHBA expression level, whereas MCF-7 cells expressed a relatively lower level of INHBA. Therefore, MCF-7 and BT549 cells were used for further experiments.

Subsequently, knocking down endogenous INHBA by shRNAs (sh-INHBA#1 and #2) in BT549 cells was able to reduce the gene and protein expression (*P* < 0.001, Figure 2b). CCK-8 cell proliferation assay and colony formation assay demonstrated that the knockdown of INHBA inhibited the growth rate and suppressed the colony forming ability in BT549 cells (*P* < 0.001, Figure 2c, d). Furthermore, the cell migration and invasion assays revealed that INHBA knockdown significantly impaired the migratory and invasive capacity in BT549 cells (*P* < 0.001, Figure 2e, f), indicating that INHBA deficiency inhibited BC cell migration.

### 3.3. INHBA overexpression enhances the invasion of BC cells

**Figure 3.**
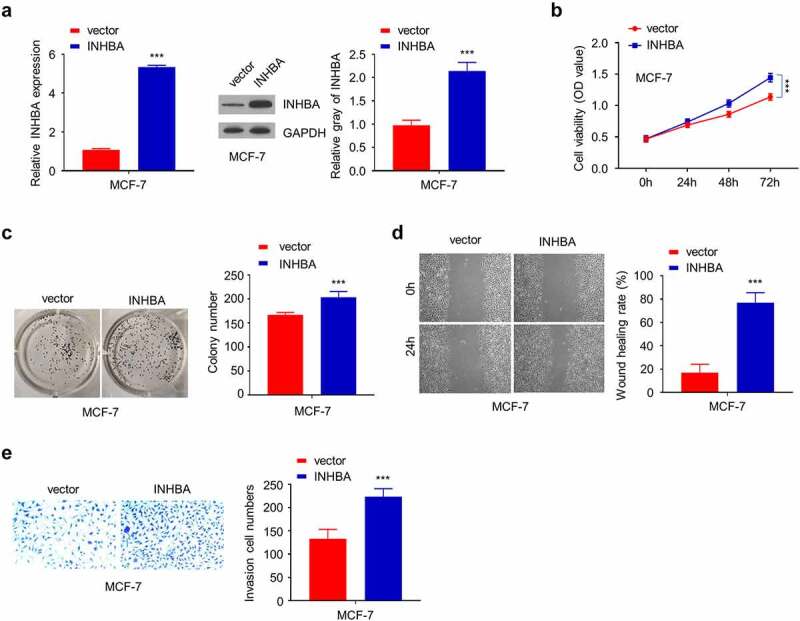
The overexpression of INHBA enhances BC cells migration and invasion. (a) The efficiency of INHBA overexpression in MCF-7 cells transfected by human INHBA cDNA or empty vectors were assessed by RT-qPCR and Western blot. GAPDH was used as a loading control in Western blot. (b) CCK-8 assay and (c) colony formation assay were performed to examine the cell proliferation of MCF-7 cells after the transfection of cDNA. (d) The migration of transfected cells was examined by wound healing assays. (e) The invasion of transfected cells was determined by transwell assays (×200 magnification). Data are summary of 3 independent experiments (n = 3). The error bars are defined as s.d. *, *P* < 0.05, **, *P* < 0.01, and ***, *P* < 0.001

In the meanwhile, transient transfection of human INHBA cDNA elevated INHBA mRNA and protein level (*P* < 0.001, Figure 3a), and CCK-8 assay and colony formation assay showed that INHBA overexpression could enhance cell growth in MCF-7 cells (*P* < 0.001, Figure 3b, c). In addition, transient INHBA overexpression in MCF-7 cells augmented the cell migration and invasion in MCF-7 cells (*P* < 0.001, Figure 3d, e), indicating that INHBA overexpression promotes BC cell invasion.

### 3.4. INHBA overexpression promotes the aggressive phenotype in non-tumorigenic epithelial cell line MCF-10A

**Figure 4.**
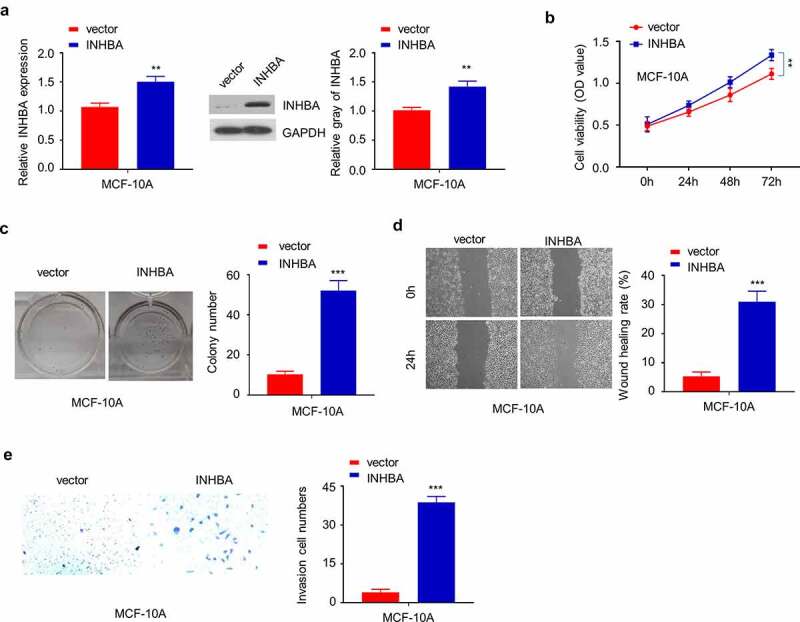
The overexpression of INHBA enhances the aggressiveness in MCF-10A cells. (a) The efficiency of INHBA overexpression in MCF-10A cells transfected by human INHBA cDNA or empty vectors were assessed by RT-qPCR and Western blot. GAPDH was used as a loading control in Western blot. (b) CCK-8 assay and (c) colony formation assay were performed to examine the cell proliferation of MCF-10A cells after INHBA overexpression. (d) The migration of transfected cells was examined by wound healing assays. (e) The invasion of transfected cells was determined by transwell assays (×200 magnification). Data are summary of 3 independent experiments (n = 3). The error bars are defined as s.d. *, *P* < 0.05, **, *P* < 0.01, and ***, *P* < 0.001

To examine whether INHBA overexpression could modulate the phenotype of non-tumorigenic cells, we transfected non-tumorigenic epithelial cell-line MCF-10A with INHBA expression vector, which significantly increased the mRNA and protein level (Figure 4a). INHBA overexpression significantly increased the cell proliferation at later stage (Figure 4b), although the effect was marginal. Other functional assay such as colony formation assay and cell migration/invasion assay also demonstrated that INHBA overexpression could enhance the aggressiveness in MCF-10A cells (Figure 4c-e). Collectively, these results indicate that INHBA overexpression could enhance the aggressiveness in non-tumorigenic cell line.

### 3.5. INHBA enhances the invasiveness of BC cells by inducing epithelial-to-mesenchymal transition

**Figure 5.**
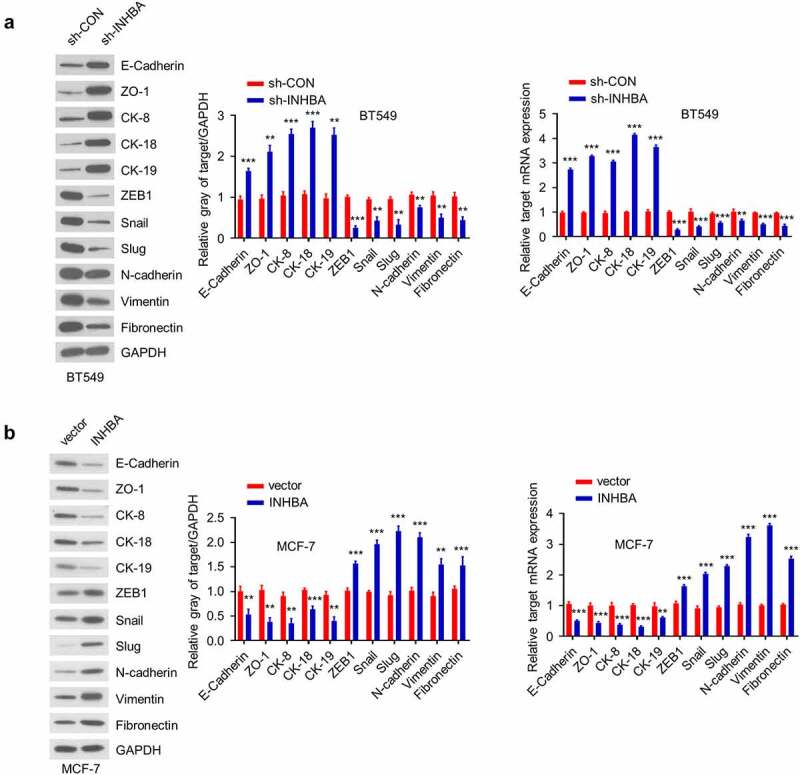
INHBA induces the EMT in BC cells. (a) Western blot analysis of the EMT-related proteins after knockdown of INHBA in BT549 cells (left) and overexpression in MCF-7 cells (right). (b) RT-qPCR analysis of the EMT-related genes after knockdown of INHBA in BT549 cells (left) and overexpression in MCF-7 cells (right). GAPDH was used as a loading control in Western blot. Data are summary of 3 independent experiments (n = 3). The error bars are defined as s.d. *, *P* < 0.05, **, *P* < 0.01, and ***, *P* < 0.001

To evaluate whether INHBA overexpression induces the epithelial–mesenchymal transition (EMT) phenotypes in BC cells, we analyzed the expression of several epithelial markers (E-cadherin, ZO-1, CK-8, CK-18 and CK-19) and mesenchymal markers (ZEB1, Snail, Slug, N-cadherin, Vimentin and Fibronectin) at the mRNA and protein levels. The expression of epithelial markers increased while the expression of mesenchymal markers decreased in BT549 cells after INHBA knockdown (*P* < 0.001, Figure 5a). On the contrary, the overexpression INHBA in MCF-7 cells reinforced the expression of mesenchymal markers but diminished the epithelial marker expression (*P* < 0.001, Figure 5b). Overall, these data suggest that INHBA induces the EMT process by promoting the phenotypical transition from epithelial state to mesenchymal state.

### 3.6. Correlation between INHBA expression and TGF-β-regulated genes and EMT-related genes in BC tissues

**Figure 6.**
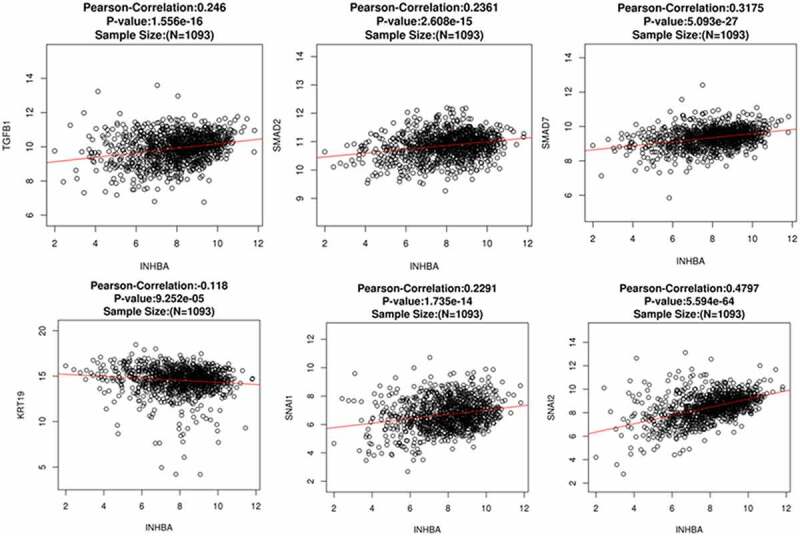
INHBA expression is positively correlated with TGF-β1, Smad2, Smad7, Snail and Slug, and negatively correlated with CK-19 in BC tissues. Pearson correlation analysis between INHBA expression and several TGF-β and EMT related genes using a web-based predictive tool ENCORI (http://starbase.sysu.edu.cn)

To further assess the correlation between INHBA level and EMT status in primary BC cells, we performed Pearson correlation analysis between INHBA expression and several TGF-β and EMT related genes using a web-based predictive tool ENCORI (http://starbase.sysu.edu.cn). The TGF-β-regulated and EMT-related genes TGF-β1, Smad2, Smad7, Snail, Slug and CK-19 (KRT19) were included for analysis. The expression of INHBA was positively correlated with the expression of TGF-β1, Smad2, Smad7, Snail and Slug in BC tissues. However, INHBA expression was negatively correlated with the expression of CK-19 (epithelial marker) in BC tissues (Figure 6). These data further indicate that INHBA expression is linked with EMT process, which may function to enhance the invasiveness of BC cells through activating the TGF-β pathways.

### 3.7. INHBA induces EMT and promotes BC cells invasion by activating the TGF-β signaling pathway

**Figure 7.**
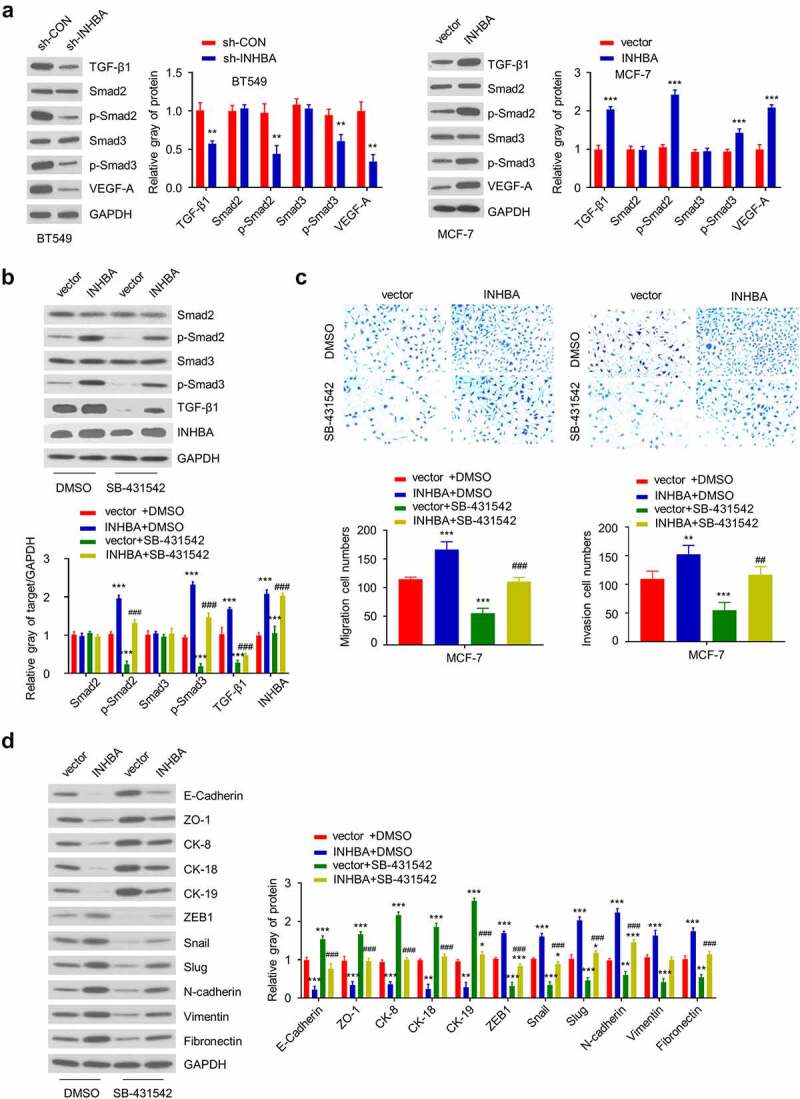
INHBA overexpression activates the TGF-β signaling pathway, which is suppressed by TGF-β inhibitor. (a) The protein level of TGF-β1, Smad2, p-Smad2, Smad3, p-Smad3 and VEGF-A in BT549 (INHBA knockdown) and MCF-7 cells (INHBA overexpression). GAPDH was used as loading control in Western blot. (b) The protein level of Smad2, p-Smad2, Smad3, p-Smad3, TGF-β1, INHBA in MCT-7 cells treated by TGF-β inhibitor (SB-431,542). 10 µM SB-431,542 was used as final concentration in the medium. DMSO solvent was also used as a negative control. (c) The cell migration (upper two) and invasion (lower two) analyses for MCF-7 cells treated with (or without) TGF-β inhibitor (×200 magnification). (d) Western blot analysis of EMT-related genes in MCF-7 cells transfected with (or without) INHBA expression vector, along with (or without) SB-431,542. Data are summary of 3 independent experiments (n = 3). The error bars are defined as s.d. *, *P* < 0.05, **, *P* < 0.01, and ***, *P* < 0.001

To functionally validate the involvement of TGF-β signaling in INHBA-induced EMT, we first examined the effect of INHBA knockdown or overexpression on activation of TGF-β signaling pathway by analyzing TGF-β1, Smad2, phosphorylated Smad2 (p-Smad2), Smad3, phosphorylated Smad3 (p-Smad3) and VEGF-A protein level by Western blotting. As shown in Figure 7a, INHBA knockdown in BT549 cells downregulated the expression of TGF-β1, p-Smad2, p-Smad3, and VEGF-A, whereas INHBA overexpression in MCF-7 cells upregulated their expression. However, the expression of total Smad2 and Smad3 remain unchanged, which indicates that INHBA regulates TGF-β signaling activation by inducing the phosphorylation of Smad2 and Smad3.

Next, to demonstrate the necessity of TGF-β signaling activity in INHBA function, an TGF-β signaling inhibitor was applied on MCF-7 cells with INHBA overexpression. MCF-7 cells transfected with INHBA expression vector or empty vector were treated with or without a specific TGF-β inhibitor (SB-431,542) for 24 hours. Then, the protein level of Smad2, p-Smad2, Smad3, p-Smad3, TGF-β, and INHBA was quantified by western blot. The results demonstrated that SB-431,542 can effectively inhibit TGF-β pathway activation in MCF-7 cells transfected with empty vectors (*P* < 0.001, Figure 7b, green bar). In addition, TGF-β inhibitor also attenuated the phosphorylation of Smad2 and Smad3 induced by INHBA expression (*P* < 0.001, Figure 7b). However, the total Smad2 and Smad3 levels were still unaffected.

Moreover, as we showed earlier that INHBA overexpression increased the MCF-7 cell migration and invasion, the addition of SB-431,542 could impair the INHBA-induced cell migration and invasion in MCF-7 cells (*P* < 0.001, Figure 7c). In addition, we showed that INHBA overexpression induced the expression of EMT-related proteins in MCF-7 cells. However, the addition of SB-431,542 significantly suppressed the upregulation of these EMT-related proteins induced by INHBA overexpression, and the suppression of epithelial proteins by INHBA overexpression was also partially rescued (*P* < 0.001, Figure 7d). Collectively, these results demonstrated that INHBA induces EMT process by activating the TGF-β signaling pathway in BC cells.

### 3.8. INHBA overexpression promotes while INHIBA silencing suppresses BC tumorigenesis

**Figure 8.**
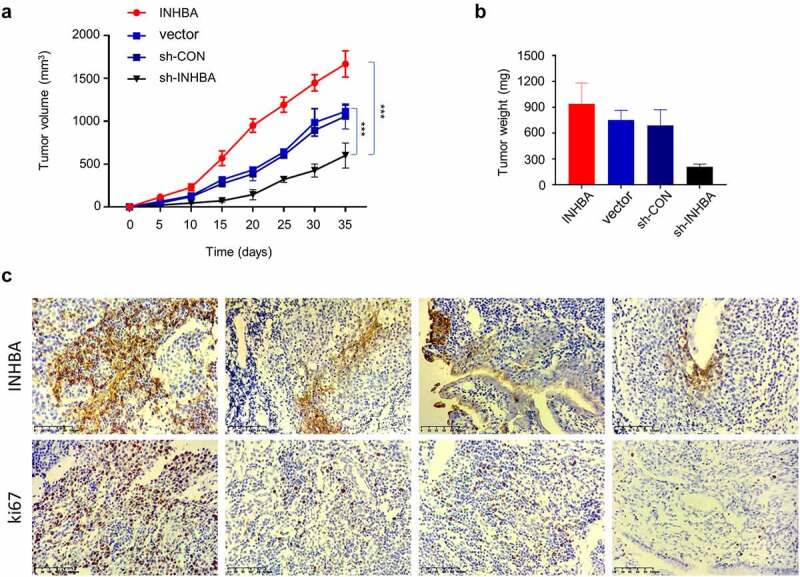
*In vivo* tumorigenesis assay corroborates the oncogenic role of INHBA in BC cells. (a-b) MCF-7 cells transfected with empty vector, INHBA cDNA, sh-INHBA, or sh-NC was injected into RAG1-deficient mice (n = 6 mice in each group). The tumor volume and weight were measured for 7 weeks after tumor cell injection. (c) IHC staining of Ki-67 (cell proliferation marker) and INHBA in the tumor tissues of different experimental groups

To further validate the role of INHBA in tumorigenesis, we injected MCF-7 cells with INHBA overexpression or INHIBA silencing into RAG1-deficient mice. The tumor volume and weight were measured for 7 weeks after tumor cell injection. We observed that the tumor growth was significantly suppressed in the INHBA silencing group, while INHBA overexpression accelerated tumorigenesis (Figure 8 A, B). Furthermore, IHC staining showed that both Ki-67 (cell proliferation marker) and INHBA were upregulated in the tumor tissues with INHBA overexpression (Figure 8c), and downregulated in the tumor tissues after INHBA silencing (Figure 8c). These data further corroborate the oncogenic role of INHBA in BC cell tumorigenesis.

## 4. Discussion

Understanding the molecular mechanisms underlying BC carcinogenesis, progression and metastasis is essential for the development of more effective treatment for BC patients [25–28]. Since EMT plays a non-trivial role in promoting metastatic growth in BC [29,30], in this study, we explored the role and mechanism of INHBA in controlling EMT of BC cells.

In our study, we first showed that INHBA overexpression was correlated with a poor prognosis in BC patients. Consistently, INHBA was significantly upregulated in BC cells. The upregulation of INHBA was also reported in other cancers by previous studies, such as gastric cancer, esophageal adenocarcinoma and colorectal cancer [11,13,16,31,32]. Wang et al. found that at least twofold increase of INHBA expression was detected in gastric tumor tissue as compared to the adjacent normal tissue, and INHBA-negative patients had a better overall survival and progression-free survival [16]. Furthermore, Bashir et al. also observed a significant increase of INHBA expression in breast tumor tissues, and INHBA expression level was inversely correlated with the overall survival of BC patients [21]. More importantly, they demonstrated that activin A, a INHBA homodimer, promotes the EMT and invasion of BC, which suggests that INHBA may be involved in metastasis of BC progression [21]. Together, our results and previous studies indicate that INHBA may function as a tumor-promoting factor in a variety of cancers.

Next, we found that INHBA overexpression promoted BC cell proliferation and migration, and in contrast, the depletion of INHBA inhibited cell viability, proliferation and migration. And these findings were validated using *in vivo* experiments. A previous study by Chen et al. also found that INHBA silencing suppresses the migration and proliferation of gastric cancer cell, and inhibited the gastric tumorigenesis in nude mice [12]. Okano et al. revealed that a high level of INHBA expression promoted the proliferation of colorectal cancer cells *in vitro* [13]. Silencing INHBA also inhibited the proliferation and invasion ability of nasopharyngeal carcinoma SUNE1 cells *in vitro* [33]. Furthermore, Kim et al. identified INHBA as one of the core metastasis-associated genes in multi-cancer analysis [34]. They showed that the overexpression of INHBA is implicated in the stromal desmoplastic reaction during the invasive transition of multiple cancers including BC [34]. These findings are consistent with the fact that activin A, a INHBA homodimer, is a TGF-β superfamily member agonist ligand and TGF-β singling activation promotes EMT and cell migration [6,7].

It is well established that cell motility and cellular plasticity could be regulated by the EMT process [29]. To further validate the functional involvement of INHBA in EMT, we compared the EMT-related gene expression in BC cells after INHBA knockdown or INHBA overexpression. We observed the transition of signature gene expression from an epithelial state to a mesenchymal phenotype after INHBA overexpression. During EMT, a switch from cell junction-related integrins to cell-extracellular matrix adhesion-related integrins has been observed [18,19,35–37]. Three transcription factors, namely Twist, Snail/Slug, and ZEB1/2, have been widely reported to regulate this switch [38]. Our study showed an increased expression of mesenchymal biomarkers (ZEB1, Snail, Slug, N-cadherin, Vimentin and Fibronectin) and a decreased expression of epithelial biomarkers (ZO-1, E-cadherin, CK-8, CK-18 and CK-19) after INHBA overexpression, which indicate that INHBA overexpression could induce the EMT process in BC cells.

Finally, we confirmed that INHBA overexpression upregulated TGF-β related genes (TGF-β1, p-Smad2, p-Smad3 and VEGF-A), and INHBA silencing impaired their expression, which implies that INHBA modulates EMT via TGF-β signaling pathway. In addition to EMT regulation, previous studies have shown that TGF-β is a pleiotropic cytokine which has immunoregulatory functions. For example, it may have a potential beneficial effect on controlling the progression of autoimmune diseases such as multiple sclerosis [39], and TGF-β also plays a pathogenetic role in fibrosis and cancer development [40,41]. These studies warrant future investigation of INHBA in autoimmune diseases and fibrotic diseases. Furthermore, a previous study has shown that activator protein-1 (AP-1) inhibitor, such as SR11302, reduces neuroinflammation and brain edema [42]. Since AP-1 transcriptionally activates INHBA expression, it is possible that AP-1 inhibitors could also inhibit INHBA expression. It is therefore plausible to speculate that the application of AP-1 inhibitor to overcome the tumor-prompting effect in BC.

It seems that TGF- β not only induces the tumorigenesis of BC at its early stage, but also promotes BC metastasis through EMT [43,44]. Upon TGF-β binding, the activation of surface receptors TβR1 and TβR2 phosphorylate and activate Smad2 and Smad3, which in turns upregulates EMT transcription factors such as Snail/Slug, ZEB1/2, and Twist [22,45]. Indeed, Chen et al. found that INHBA silencing impairs the metastasis of gastric cancer cells by negatively regulating TGF-β signaling pathway [12]. Collectively, our data and previous studies support the notion that INHBA upregulation in cancer promotes EMT and cell invasion by activating TGF-β pathway. Since TGF-β signaling pathway has a well-established role in EMT and INHBA is a secretory extracellular factor mediating TGF- β signaling, INHBA could be easily targeted by neutralizing antibody to inhibit EMT in breast cancer, which could be a novel therapeutic target especially for breast cancers with enhanced EMT phenotype.

## 5. Conclusion

Our results suggest that INHBA dysregulation contributes to the EMT and invasive properties of BC cells by activating the TGF-β signaling. Together with previous studies, we propose that targeting INHBA/TGF-β pathway may represent a potential strategy target to impede BC progression and metastasis.

## Data Availability

The datasets used and/or analyzed during the current study are available from the corresponding author on reasonable request.

## Highlights

•INHBA was un unfavorable factor upregulated in BC (breast cancer) tissues and cells.

•INHBA overexpression promoted BC cell growth and invasion by inducing EMT.

•INHBA induced EMT by activating TGF-β-regulated genes.
